# A Presurgical Study of Curcumin Combined with Anthocyanin Supplements in Patients with Colorectal Adenomatous Polyps

**DOI:** 10.3390/ijms222011024

**Published:** 2021-10-13

**Authors:** Irene Maria Briata, Laura Paleari, Mariangela Rutigliani, Marilena Petrera, Silvia Caviglia, Paola Romagnoli, Mauro Dalla Libera, Massimo Oppezzi, Matteo Puntoni, Giacomo Siri, Matteo Lazzeroni, Lynne Howells, Raj Singh, Karen Brown, Andrea DeCensi

**Affiliations:** 1 Division of Medical Oncology, E.O. Galliera Hospital, 16128 Genoa, Italy; irene.maria.briata@galliera.it (I.M.B.); Marilena.petrera@galliera.it (M.P.); 2 Research, Innovation and HTA, A.Li.Sa. Liguria Health Authority, 16121 Genoa, Italy; 3 Division of Pathology, E.O. Galliera Hospital, 16128 Genoa, Italy; Mariangela.rutigliani@galliera.it (M.R.); Giacomo.siri@galliera.it (G.S.); 4 Clinical Trial Unit, Office of the Scientific Director, E.O. Galliera Hospital, 16128 Genoa, Italy; silvia.caviglia@galliera.it; 5 Division of Gastroenterology, E.O. Galliera Hospital, 16128 Genoa, Italy; Paola.romagnoli@galliera.it (P.R.); Mauro.dallalibera@galliera.it (M.D.L.); Massino.opezzi@galliera.it (M.O.); 6 Clinical & Epidemiological Research Unit, University Hospital of Parma, 43126 Parma, Italy; untoni@inwind.it; 7 Division of Cancer Prevention and Genetics, IEO European Institute of Oncology IRCCS, 20141 Milan, Italy; Matteo.lazzeroni@galliera.it; 8 Leicester Cancer Research Centre, University of Leicester, Leicester LE1 7RH, UK; lh28@le.ac.uk (L.H.); rs25@le.ac.uk (R.S.); kb20@le.ac.uk (K.B.); 9 Wolfson Institute of Preventive Medicine, Queen Mary University of London, London E1 4NS, UK

**Keywords:** curcumin, anthocyanins, adenomatous polyps, dietary supplement, Ki-67 antigen, NF-kappa B, prevention

## Abstract

Adenomatous polyps are precancerous lesions associated with a higher risk of colorectal cancer (CRC). Curcumin and anthocyanins have shown promising CRC-preventive activity in preclinical and epidemiological studies. The objective of this window-of-opportunity, proof-of principle trial was to evaluate the effect of curcumin combined with anthocyanin supplements on tissue biomarkers of colorectal adenomatous polyps. Eligible patients received either anthocyanin and curcumin supplementation or related matching placebo for 4–6 weeks before polyp removal. Adenomatous polyps and adjacent tissue biopsies were collected at baseline and after supplementation for immunohistochemical assessment of β-catenin, NF-kappa B (NF-κB), Ki-67, P53, and dysplasia. No differences were observed in baseline biomarker expression between normal and dysplastic tissues. The combination of anthocyanins and curcumin resulted in a significant borderline reduction of NF-κB immunohistochemistry (IHC) expression in adenoma tissue (geometric mean ratio (GMR): 0.72; 95% confidence interval (CI): 0.51–1.00; *p*-value: 0.05) and a trend to a reduction of Ki-67 (GMR: 0.73; 95% CI: 0.50–1.08; *p*-value: 0.11). No significant modulation of biomarkers in normal adjacent mucosa was observed. We concluded that the combined supplementation of anthocyanins and curcumin seems to lead to a potentially favorable modulation of tissue biomarkers of inflammation and proliferation in colon adenomas.

## 1. Introduction

Colorectal cancer (CRC) is the second most commonly diagnosed malignancy and one of the main causes of cancer death in both men and women [1]. CRC is a multifactorial disease with approximately 60–70% of sporadic cases [2]. The main risk factors are age, family history, the presence of adenomatous polyps (APs), diffuse polyps, and the presence of hereditary conditions such as Lynch syndrome or familial adenomatous polyp (FAP) syndromes.

APs are present in approximately one-half to two-thirds of patients diagnosed with colorectal polyps. APs are precancerous lesions associated with a higher risk of CRC, particularly the advanced ones (≥1 cm in diameter, villous histology, or high-grade dysplasia) with or without multiplicity (>3 adenomas) [2]. The adenoma–carcinoma progression happens over approximately 10 years; therefore, APs are considered a reliable intermediate marker (surrogate) of risk and efficacy for chemoprevention studies [3]. 

In the colon, besides genetic mutations that may promote the initiation of carcinogenesis (e.g., mutation in *APC*, *k-RAS*, and *P53*), cytokine, and reactive oxygen species (ROS) production due to the fact that inflammation can lead to mutations and the progression of adenoma to carcinoma [4]. Accordingly, treatment with non-steroidal anti-inflammatory agents has been shown to reduce long-term CRC incidence and mortality [5]. The development of CRC is highly influenced also by modifiable environmental factors such as lifestyle and dietary habits. A growing body of evidence has reported that a diet rich in proinflammatory components, such as red and processed meat, and poor in fruits and vegetables, is associated with a higher risk of developing CRC [6,7]. Several plant-derived components have shown anti-inflammatory and antioxidant activities and have been studied for their role in cancer prevention. Curcumin is the main biologically active compound derived from the roots of *Curcuma longa* and is commonly utilized as a spice, especially in Asia. This natural polyphenol shows antioxidant and anti-inflammatory effects through the regulation of numerous intracellular targets that control CRC cell growth in in vitro and in vivo studies [8]. On the other hand, clinical studies in humans are very few and have shown conflicting results [8,9]. A wide range of curcumin formulations have been explored during recent years in order to improve its curcumin poor bioavailability and low oral absorption. In this scenario, the food-grade formulation of curcumin in phospholipids (Meriva^®^) has been demonstrated to improve curcumin absorption [10] and led to a higher production of curcuminoid catabolites than unformulated curcumin extract thanks to microbial biotransformation [11]. Curcumin phospholipids have shown stronger hepatoprotective, anti-inflammatory and chemopreventive effects in a preclinical study through the activation of PPARγ and the inhibition of NF-kappa B (NF-κB) than unformulated curcumin [12]. Furthermore, curcumin phytosome has been demonstrated to be effective in several unhealthy conditions keeping a good safety profile, as reported in several human studies [13,14,15].

Another dietary component with demonstrated biological activity consistent with a proven anticancer effect in preclinical models are anthocyanins, natural flavonoids responsible for the red, blue, and purple colors of numerous fruits and vegetables. The phenolic structure of anthocyanins is responsible for their antioxidant and anti-inflammation activity, mainly due to their ability to scavenge ROS [16]. In addition, it was demonstrated that anthocyanins can inhibit CRC, interfering in the cell cycle and inducing antiproliferative effects and apoptosis [17,18,19]. Data from nonrandomized controlled trials demonstrate that anthocyanins may play an active role in the prevention of CRC in humans [19]. Mirtoselect^®^ is one of many commercially available anthocyanin extracts; it is a standardized bilberry extract derived from *Vaccinium myrtillus L.* and contains 36% (*w*/*w*) anthocyanins, including the predominant anthocyanin constituents, such as the 3-galactoside, 3-glucoside, and 3-arabinoside of delphinidin and the 3-galactoside and 3-glucoside of cyanidin. The beneficial effects of this bilberry extract are supported by several studies in humans [20,21,22]. Moreover, Mirtoselect has a highly specific fingerprint, identified through HPLC analysis for guaranteeing the extract quality and is tested by DNA analysis during quality-control procedures to ensure the quality, traceability, and authenticity of the material.

In the present clinical trial, we aimed to evaluate the safety and biological effects of the prolonged supplementation of curcumin (Meriva) combined with an anthocyanin extract (Mirtoselect) on tissue biomarkers of colorectal APs.

## 2. Results

**Figure 1:**
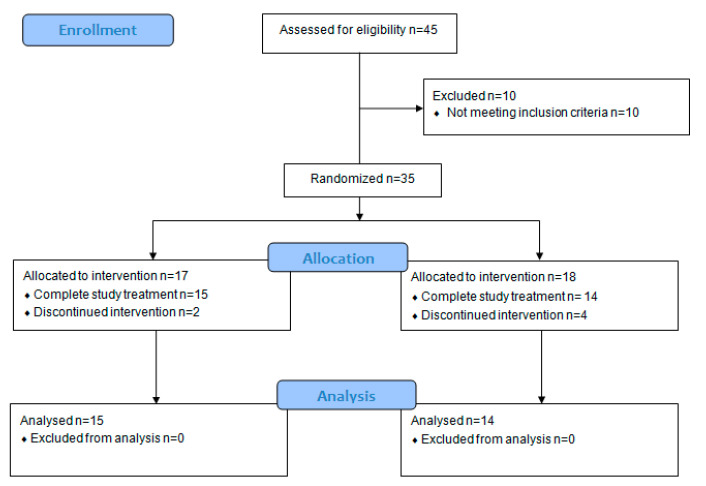
CONSORT flow diagram of the phases of the MiRACol randomized trial of the active and placebo group as well as the number of patients included in the analysis.

Between March 2014 and December 2017, 45 patients were enrolled in the MiRACol study (NCT01948661): 35 were randomized, while 10 did not meet the eligibility criteria mainly because of the absence of polyp with ≥1 cm in diameter. Six participants withdrew their study consent, with 2 in the active arm and 4 in the control group, and were excluded from the analysis. The trial flow-chart is depicted in Figure 1. 

Participants and disease characteristics at baseline were well balanced between the two arms, as reported in Table 1. More specifically, 10 (66.7%) participants enrolled in the active group were male and 5 (33.3%) were female, whereas an equal gender distribution (7.50% male and 7.50% female) was apparent in the control group between subjects who completed the study. No difference in the mean age of patients was observed (70.8 ± 9.8 vs. 67.9 ± 10.8 years; *p*-value: 0.454). The majority of the patients had a high level of education. No differences between groups were observed in smoking habits: 53.3% of participants in the active arm and 50% of patients in the control group were former smokers. A consumption of 2 or more glasses per meal of alcohol-containing drinks was recorded in 66.7% and 50% of the patients enrolled in the active and control arms, respectively. The baseline body mass index (BMI) was slightly higher in the active arm than in the control group (≥25 BMI: 53.3% vs. 35.7%). The dietary intake of food rich in anthocyanins was collected through a food diary. No difference in the consumption of food rich in anthocyanins was observed between groups. The patients were advised not to use curcumin as a spice (in the form of turmeric) or supplement during the study; therefore, the data on its use were not collected. Adenomas were mostly classified as low-grade (80% in the active arm; 64.3% in the control arm) with tubular histology (66.7% in the active arm; 71.4% in the control group). Although there were no differences in the number of comorbidities (interquartile range (IQR): 0–3 in the active arm and 1–3 in the control group), the participants enrolled in the control group used a larger number of concomitant medications (IQR: 0–4 in the active arm and 2–5 in the control group; *p*-value: 0.086).

Compliance was checked by a self-reported diary and a pill count. The overall compliance was very high, as 100% of the patients showed a pill intake higher than 80%.

No serious adverse events were observed during the 4–6 weeks of supplementation. The incidence of AE was low and mainly due to gastrointestinal disturbances such as dyspepsia (2/10; 20%), stomach pain (1/10; 10%), nausea (1/10; 10%), and diarrhea (1/10; 10%). These mild AEs were not related to the active supplemented combination; thus, no differences in AEs between active or placebo arms were detected (data not shown).

**Figure 2:**
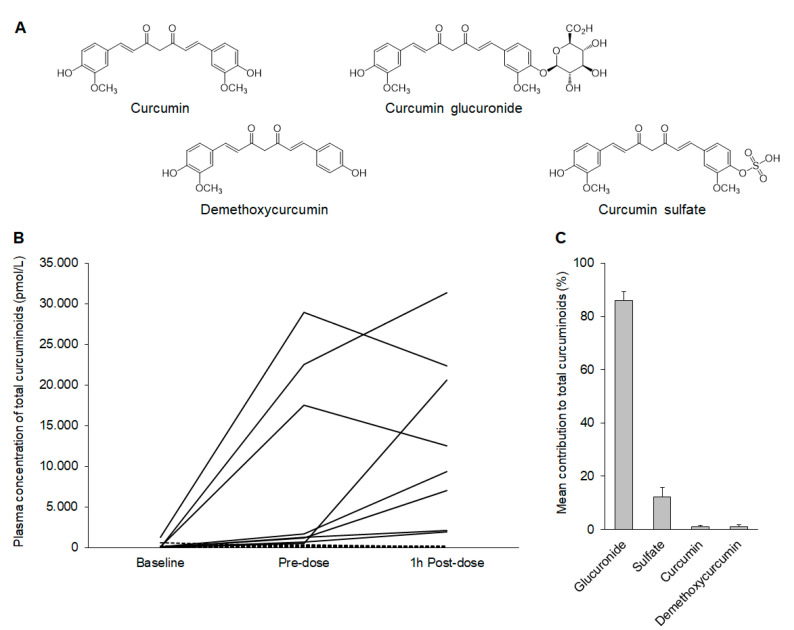
Analysis of plasma curcuminoids in samples collected at baseline and on two occasions on the final day of the study, immediately prior to the last dose and 1 h post-dose. Only a subset of patients were selected at random for LC-MS/MS analysis. (**A**) Structures of curcumin, demethoxycurcumin, and the mono-conjugated sulfate and glucuronide metabolites analyzed in the samples. (**B**) Plasma concentrations of the total curcuminoids in each patient at the three time points. Solid lines correspond to the patients in the active arm (n = 8), and the dashed lines indicate the patients in the placebo arm (n = 7). (**C**) The profile of curcumin species detected in the patients in the active arm 1 h post-dose. Values shown are the mean (±SEM) contribution of each curcumin species detected to the total quantifiable curcuminoids for the 8 patients. The profile was similar in samples collected immediately prior to the last dose (not shown).

Plasma samples collected at baseline before starting the intervention and during the last study visit when patients consumed their final doses were analyzed for the presence of parent curcumin and its major metabolites (Figure 2A, Supplementary Materials Figures S1 and S2). There was a clear increase in total plasma curcuminoids above baseline for all patients in the active arm, with concentrations reaching the low nM range (Figure 2B). Glucuronide conjugates were the predominant species detected in samples taken both immediately prior to and 1 h post-dose, accounting for ~85–90% of the total curcuminoids in the patients in the active arm (Figure 2C). Sulfate metabolites were also present in appreciable amounts, whereas parent curcumin represented just ~1–2% of the curcuminoids detected; these finding are consistent with the well-recognized rapid metabolism of curcumin in humans [23,24]. 

### Tissue Biomarker Expression

Ki-67, P53, NF-κB, and β-catenin protein expression levels were evaluated in both adenomatous and normal tissues in both active and placebo arms. The biomarker expression levels were comparable between active and placebo arms; no differences were observed in the expression of any of the biomarkers when comparing both at baseline and after supplementation. The results are reported in Table 2. 

**Figure 3:**
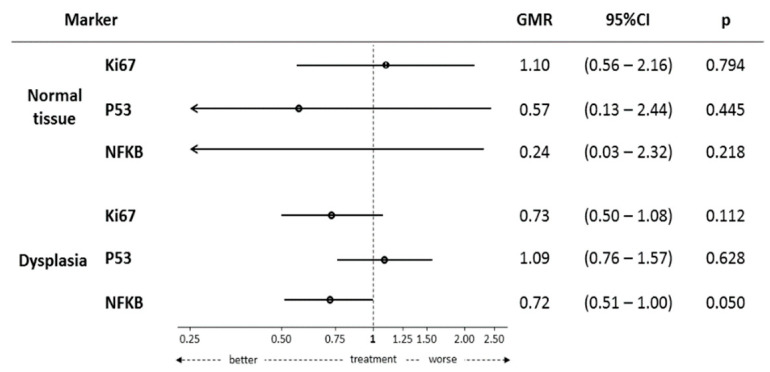
The effect of treatment on tissue biomarkers is assessed as a GMR. GMR is obtained from a generalized linear model with the log-transformed value of each marker with outcome and treatment (active vs. placebo) as the predictors. Each model is adjusted for the log-transformed baseline value of the marker, age, and sex. Other possible covariates (see Table 1) are inserted, if they involve a change greater than 5% on the GMR related to the effect of treatment. Abbreviations: Ki-67, protein encoded by the MKI67 gene; P53, protein encoded by the TP53 gene; NF-κB, nuclear factor kappa-light-chain-enhancer of activated B cells; β-catenin, protein encoded by the CTNNB1 gene; GMR, geometric mean ratio; CI, confidence interval.

Figure 3 depicts the effects of the curcumin and bilberry extract combination on various biomarker levels. When comparing pre- and post-treatment levels within each group, the combination of anthocyanins and curcumin resulted in a borderline significant reduction of the NF-κB expression, assessed by immunohistochemistry (IHC), in adenoma tissue (geometric mean ratio (GMR): 0.72; 95% confidence interval (CI): 0.51–1.00; *p*-value: 0.05) and a trend towards a reduction for Ki-67 (GMR: 0.73; 95% CI: 0.50–1.08; *p*-value: 0.11). In contrast, no significant modulation of biomarkers in normal adjacent mucosa was observed.

**Figure 4:**
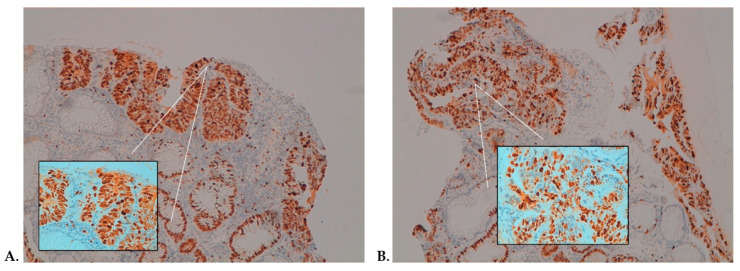
Immunohistochemistry (IHC) assessment of Ki-67 in high-grade dysplasia showing positive staining in 100% of cells in the pre-treatment sample (**A**) and 80% of cells post-treatment (**B**) (magnification: 10× and 40× in the box). Panels A and B are representative of the pre- and post-treatments from the same patient.

**Figure 5:**
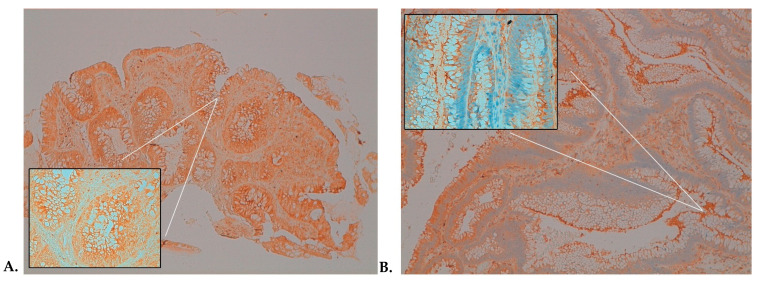
Immunohistochemical assessment of NF-κB-p65 protein in high-grade dysplasia showing positive cytoplasmic staining in 80% of cells in pre-treatment tissue sample (**A**) and 60% of cells in post-treatment samples (**B**) (magnification: 10× and 40× in the box). Panels A and B are representative of the pre- and post-treatments from the same patient.

In almost the 54% of patients allocated to the treatment arm, a favorable modulation of Ki-67 and NF-κB between pre- and postsupplemented specimens was observed. Here, we reported two representative examples belonging to the same patient with a high-grade adenoma: Figure 4 shows a 20% reduction of the Ki-67 expression, and a similar 20% reduction was observed for NF-κB-p65 (Figure 5).

## 3. Discussion

Curcumin and anthocyanin extracts have been demonstrated to be phytochemicals with potential antitumor activity in CRC [8,9,16,17,18,19]. A recent systematic review shows a significant number of clinical trials conducted with curcumin formulations in a cancer-preventive setting with positive outcomes, but only 30% include putative biomarkers of efficacy as clinical endpoints [9]. Conversely, data relating to the CRC-preventive activity of anthocyanins are mainly based on observational studies [19]. Therefore, it is difficult to translate promising preclinical findings into clinical settings with a potential benefit for patients. In our study, we investigated the effect of the combination of anthocyanins with curcumin phospholipids formulation on the modulation of tissue biomarkers in patients with the precancerous lesion of the colorectal tract. To our knowledge, this is the first clinical study investigating this combination in the context of colorectal adenomas. We found that the combined concomitant supplementation resulted in a possibly favorable modulation of NF-κB and Ki-67 in colorectal adenomas, suggesting that both inflammation and proliferation are moderately inhibited by the combination. The role of NF-κB in cancer promotion and progression is well-known, since its activation can control cancer cell proliferation by activating genes of growth factors and apoptosis. Therefore, suppressing the NF-κB signaling pathway could represent an interesting strategy for cancer prevention [25]. Previous in vitro studies have revealed that curcumin can inhibit cell growth by blocking the cell cycle and promoting apoptosis through several mechanisms including the inhibition of transcription factors, such as NF-κB and β-catenin, and the production of ROS [8]. Curcumin can also inhibit the nuclear translocation of the NF-κB subunit [26] and cytokine-mediated NF-κB activation by blocking a signal leading to IKK activity [27]. In addition to the antioxidant ability of anthocyanins, some preclinical studies have shown that anthocyanins may induce apoptosis and inhibit cell proliferation through blocking various phases of the cell cycle [17,18,19]. A previous study in CRC patients showed that seven consecutive days of supplementation with the same anthocyanin-rich extract from bilberry can reduce the proliferation index in tumors by 7%, as reflected by Ki-67 staining (*p* = 0.003), compared with the pre-intervention values [28]. Our hypothesis is that the combined supplementation of anthocyanins and curcumin phospholipid formulation might inhibit the NF-κB signaling pathway directly or through the reduction of ROS production and therefore decrease cell proliferation. We showed that the antiproliferative effect of the combined supplementation was significant only in cancer cells; these results are consistent with previously data suggesting a selective effect on the growth of cells [19]. 

A good safety profile was also observed along with an adequate adherence to supplementation as shown by the plasmatic levels measured in the patients’ subset. The prevalence of conjugated form for polyphenols represents a “clever” biomimetic delivery system of parent compounds to inflamed tissues, as already discussed by Perez-Vizcanio [29] and Shimoi K [30].

This is an encouraging finding, which provides the background for further studies in the nutraceutical field. Furthermore, the presurgical window study proved to be feasible even in a precancerous setting and allowed the assessment of the effect of curcumin and anthocyanins in a real target tissue for preventive activity. This is a strength of the current trial, because often window studies are performed in patients with cancer, as it is relatively easier to obtain surplus tissue from surgical resections of malignant tumors. Indeed, most of the previous similar studies with nutraceuticals were conducted on cancer patients [8,28,31]. Here, we exploited the fact that, for some patients, polypectomy cannot be performed during colonoscopy due to the dimensions of the adenoma or risk of bleeding, resulting from not discontinuing anticoagulant drugs prior to the procedure.

A further advantage of this trial design was that the window between biopsy and surgery allowed us to treat the participants for 4–6 weeks whereas analogous studies in cancer patients would typically be limited to a much shorter duration. Limitations of the current study include the lower-than-anticipated accrual, mostly due to the logistical organization and the perception of high patient burden for the double colonoscopy. Moreover, we failed to see a modulation of β-catenin, which was our primary endpoints. Another limitation is the highly variability of some biomarkers including NF-κB and p53 due to the low number of observation.

In conclusion, the combined supplementation of anthocyanins and curcumin phospholipid formulation seems to lead to a potentially favorable modulation of tissue biomarkers of inflammation and proliferation in colon adenomas, although the small sample size of patients enrolled in the present study. Thus, further studies are warranted to confirm our results and to hypothesize a positive effect of this supplementation for the prevention of CRC in high-risk individuals. 

## 4. Materials and Methods 

### 4.1. Study Design and Participants

We conducted a randomized, double-blind, placebo-controlled, phase II presurgical trial in patients with APs of the colon who receive either anthocyanin and curcumin or placebo for 4–6 weeks before polypectomy. The eligibility criteria included an age range between 18 and 85 years, the presence of one or more APs in the colorectal tract with a diameter of ≥1 cm, and ECOG Performance Status of ≤1. The exclusion criteria were the presence of hyperplastic polyps, flat serrated adenomas, or cancer. Patients who had taken experimental medications or bilberry-based dietary supplements or curcumin in the 15 days prior to enrollment were also excluded. 

Participants were recruited from the Department of Gastroenterology at Galliera Hospital (Genoa, Italy), between March 2014 and December 2017. 

The study (NCT01948661) was approved by the local Ethical Committee (Comitato Etico Regione Liguria) and conducted in accordance with Good Clinical Practice and the Declaration of Helsinki. All patients provided written informed consent prior to participation.

### 4.2. Study Procedures

During the colonoscopy, tissue biopsies of the polyp and peri-lesional normal tissue were collected. After histological confirmation and eligibility criteria verification, the eligible patients were randomized to active treatments (active arm) or placebo (control arm). After 4–6 weeks of supplementation, adenomas were removed, and a biopsy of adjacent normal tissue was collected. The participants were asked to refrain from using curcumin as a spice and to complete a diary to report the intake of fruits and vegetables rich in anthocyanins. The tissue biomarker assessments were performed on both adenomatous and normal tissues. 

### 4.3. Dietary Supplements

Curcumin (Meriva^®^) anthocyanin (Mirtoselect^®^) and related matching placebo were kindly donated by Indena SpA (Milan, Italy). Curcumin typically exhibits poor oral absorption in the body [31]. However, Meriva is a food-grade lecithin formulation of curcumin in 500 mg film-coated tablets, containing a standardized amount of 100 mg curcuminoids. The phospholipid formulation allows an improvement in curcuminoid bioavailability by about 30 fold over the standard turmeric extracts [10,32]. The product proved to be safe and well tolerated in several clinical trials [10,13,33,34]. Mirtoselect is a standardized extract from bilberry (*Vaccinium myrtillus*) containing ≥36% of anthocyanins, including cyanidin-3-glucoside (C3G). Mirtoselect safety and efficacy have been supported by at least 30 controlled or double-blind studies on vascular and eye disorders [20,21]. Moreover, Mirtoselect is certified by DNA analysis during quality control procedures to ensure the quality, traceability, and authenticity of the extract. Tablets of 500 mg Mirtoselect were prepared. 

Placebo tablets identical to active ones in terms of size, shape, color, odor, and taste were prepared. Before release, all film-coated tablets were controlled for the appearance, the average mass, the uniformity of mass, and the HPLC content of the total active components (curcumin or anthocyanins), the disintegration time, and the microbiological quality. All procedures were performed according to Food Supplement European Regulation.

After randomization, the subjects were instructed to take 1 tablet of 500 mg Mirtoselect or related matching placebo and 1 tablet of 500 mg Meriva or related matching placebo before breakfast and dinner every day (4 tablets/daily) for 4–6 weeks. The decision to use a daily dose of 1 g blueberry extract was based on existing studies that report its safety even in greater doses [28,35]. Similarly, the decision to use 1 g of Meriva was based on previous studies, which demonstrate that the phospholipids formulation-phosphatidylcholine complex is capable of increasing the bioavailability of curcuminoids [10,31], allowing the use of lower doses compared to those in previous studies with standard unformulated curcumin [36]. Blood samples were taken at baseline before commencing the supplementation and on the final day of the study, immediately before and 1 h after the last doses of Mirtoselect^®^ and Meriva^®^ or placebo. For a proportion of randomly selected patients, plasma samples were subject to LC-MS/MS analysis using our established assay to determine the concentrations of curcumin, desmethoxycurcumin, and the major conjugated glucuronide and sulfate curcumin metabolites [37]. All the samples were coded and analyzed blind. 

### 4.4. Liquid Chromatography-Electrospray Ionization (LC-ESI)-MS/MS Analysis and Quantitation of Curcumin and Metabolites

Blood samples were taken at baseline before commencing the supplementation and on the final day of the study, immediately before and 1 h after the last doses of Mirtoselect^®^ and Meriva^®^ or placebo. For a proportion of randomly selected patients, plasma samples were subject to liquid chromatography-electrospray ionization (LC-ESI)-MS/MS analysis using our established assay outlined below to determine the concentration of curcumin, demethoxycurcumin, and the major mono-conjugated glucuronide and sulfate curcumin metabolites [37]. All the samples were coded and analyzed blind. 

The identities of curcumin species in the patient samples were determined based on identical chromatographic properties and selected reaction monitoring [M-H]^–^ ion transitions to authentic standards. Curcumin glucuronide and sulfate standards were synthesized in-house as the mono-substituted metabolites using previously published methods [36,38,39]. The standards were characterized by mass spectrometry and proton nuclear magnetic resonance (^1^H-NMR) spectroscopy and had a purity of ≥95%. Standard curcumin was obtained from Indena SpA (Milan, Italy). Curcumin preparations extracted from natural sources typically contain small amounts of demethoxycurcumin and bis-demethoxycurcumin in addition to the parent curcumin and are collectively referred to as curcuminoids.

Plasma samples (250 μL) were subject to liquid extraction using acetone/formic acid as previously described [36] and the supernatant concentrated to dryness and resuspended in 50 μL of 0.1% acetic acid/acetonitrile (0.1% acetic acid) (40:60 *v*/*v*). The samples were vortexed and then centrifuged, and the supernatant was transferred to HPLC vials for injection onto the LC-ESI-MS/MS system, which consisted of a Waters Alliance 2695 separations module and a Waters 2487 UV detector, connected to a Micromass Quattro Platinum (Waters Ltd., Borehamwood, UK) tandem quadrupole mass spectrometer with an electrospray interface. The full details of the analysis conditions and instrument settings have been reported previously [36]. For each sample, 20 µL were injected onto a HyPurity C_18_ column (2.1 mm × 150 mm, 3 µm) attached to a HyPurity C_18_ guard cartridge (2.1 mm × 10 mm, 3 µm) (Thermo Electron Corporation, Runcorn, UK) connected to a KrudKatcher (5 µm) disposable precolumn filter. The column was eluted by a gradient mobile phase of A, 0.1% acetic acid and B, and acetonitrile (0.1% acetic acid) at a flow rate of 0.2 mL/min. The gradient comprised 0 min-10%B, 15 min-40%B, 25 min-85%B, 30 min-100%B, 35 min 100%B, 35.1 min-10%B, and 45 min-10%B. The column oven temperature was maintained at 30 °C.

The samples were analyzed in negative ESI mode with selected reaction monitoring for the following [M-H]^-^ ion transitions: curcumin 367→134 and 367→149 m/z, demethoxycurcumin 337→119 m/z, curcumin glucuronide 543→217 m/z, and curcumin sulfate 447→217 m/z. Transitions corresponding to the conjugated metabolites of demethoxycurcumin were also monitored, but no peaks were observed in the patient samples. β-Estradiol was employed as an internal standard and detected using the SRM [M-H]^-^ ion transition 271→145 m/z. Data were acquired using MassLynx software (version 4.0). The concentrations of curcumin and its metabolites in plasma samples were determined using individual calibration lines constructed by the addition of different amounts of each analyte to the control sample matrix (ranging from 20 to 1000 fmol detected in the column) [36]. Total plasma curcuminoids were calculated by the summation of all curcumin species detected in each sample.

### 4.5. Tissue Biomarkers Assessment

Formalin-fixed samples were embedded in paraffin, cut at 2 μm, stained with hematoxylin and eosin and used for IHC analysis. 

Primary antibodies for IHC included rabbit antibodies against Ki-67 (clone 30–9, 1/100; Ventana), NF-κB-p65 (clone F6, pre-diluted; Santa Cruz Biotechnology, Dallas, TX, USA), β-catenin (clone 14, prediluted; Cell Marque, Rocklin, CA, USA), and p53 (clone Do-7, pre-diluted; Ventana, Big Sur, CA, USA). Heat-induced antigen retrieval was performed with a citrate buffer at pH 6 for c-Jun, cleaved caspase-3, NF-κB p65, and β-catenin or with an EDTA buffer at pH 8 for Ki-67, DKK1, and INCENP. Rabbit primary antibody binding was detected with goat anti-rabbit polymer HRP (ZytoChem Plus, Berlin, Germany). Color was developed with Diaminobenzidine substrate-chromogen (ThermoFisher Scientific/Lab Vision, Fremont, CA, USA), and tissues were counterstained with hematoxylin.

Immunostaining was performed with an autostainer (BenchMark ULTRA IHC/ISH System, Angleton, TX, USA) according to the procedure reported previously [40]. All series included positive and negative controls. For Ki-67 (Figure 3 and Figure 4) and p53, only cells with nuclear staining were scored as positive. For β-catenin, only cells with membranous or membranous and cytoplasmic staining were scored as positive. For NF-κB-p65 (Figure 4 and Figure 5), only cells with cytoplasmic staining were scored as positive.

Two independent investigators (MR and RB), blinded to the treatment arm, patients’ characteristics, and the time of tumor sampling, evaluated the immunostaining results. The semi-quantitative analysis was applied to score Ki-67 expression: for each sample, the percentage of positive dysplastic cells among the total number of tumor cells was evaluated. Moreover, all the positive cells for the tissue biomarkers assessed by IHC expressed an intensity from moderate (++) to severe (+++).

### 4.6. Statistical Analysis

Descriptive statistics were provided for all variables in the summary tables by treatment group according to the type of variable summarized. Continuous variables were summarized by using n (sample size), geometric mean, and coefficient of variation (CV) or arithmetic mean and standard deviation (SD) according to their distribution, median, and percentiles. Categorical variables were summarized by using frequency distributions and percentages. Univariate tests were performed to explore the distribution of each variable according to the treatment group and the time point. *t*-tests or Kruskal–Wallis tests were adopted for continuous variables, while Fisher’s Exact or Chi-squared tests were used for categorical variables. The main analysis on the effect of treatment at polypectomy was conducted using several generalized mixed models on the log-transformed value of each marker. Each model was adjusted for the baseline value of the marker, age, and sex. Other possible covariates were inserted, if they involved a change greater than 5% on the GMR related to the effect of treatment. Hypothesis testing was carried out at the alpha = 0.05 level (two-sided) when comparing treatments. Statistical significance was declared, if the rounded *p*-value was be less than or equal to 0.05. The analysis was performed using STATA (StataCorp. 2015. Stata Statistical Software: Release 14.2. StataCorp LP, College Station, TX, USA).

## Figures and Tables

**Table 1:** Patients’ baseline characteristics by treatment arm.

Parameter		Treatment		*p*-Value
		Active	Placebo	
N (%)		15 (51.7)	14 (48.3)	
Days of treatment, mean ± SD		37.7 ± 5.4	37.4 ± 4.9	0.873
Gender, n (%)	F	5 (33.3)	7 (50.0)	0.462
	M	10 (66.7)	7 (50.0)	
Age, mean ± SD		70.8 ± 9.8	67.9 ± 10.8	0.454
Level of education, n (%)	Primary–middle	5 (33.3)	6 (42.9)	0.710
	High university	10 (66.7)	8 (57.1)	
Smoker, n (%)	No	4 (26.7)	5 (35.7)	1.000
	Former	8 (53.3)	7 (50.0)	
	Yes	3 (20.0)	2 (14.3)	
Alcohol habits, n (%)	No	5 (33.3)	7 (50.0)	0.462
	Current/former	10 (66.7)	7 (50.0)	
Baseline BMI, n (%)	<25	7 (46.7)	9 (64.3)	0.462
	≥25	8 (53.3)	5 (35.7)	
Family history of CRC, n (%)	No	10 (66.7)	7 (50.0)	0.462
	Yes	5 (33.3)	7 (50.0)	
Foods rich in anthocyanins, (servings per day)	<2	7 (46.7)	7 (50.0)	1.000
	≥2	7 (53.3)	7 (50.0)	
Histological type, n (%)	Tubular	10 (66.7)	10 (71.4)	1.000
	Villous	5 (33.3)	4 (28.6)	
Dysplasia grade, n (%)	Low-grade	12 (80.0)	9 (64.3)	0.427
	High-grade	3 (20.0)	5 (35.7)	
# of comorbidity	median (IQR)	1 (0–3)	2 (1–3)	0.295
# of concomitant medications	median (IQR)	2 (0–4)	4 (2–5)	0.086

Data are presented as number (%), except those that were differently noted. Abbreviations: SD, standard deviation; F, female; M, male; BMI, body mass index; CRC, colorectal cancer; IQR, interquartile range; #, number.

**Table 2:** Tissue biomarker expression according to the treatment arms and the time points in normal and dysplastic tissues.

		Treatment		*p*-Value
		Active	Placebo	
N (% of Positive Cells)		15 (51.7)	14 (48.3)	
Normal Tissue				
Ki-67%	Pre	19.4 ± 13.1 14.6 (0.7)	21.2 ± 11.4 18.6 (0.5)	0.565
	Post	16.1 ± 11.8 11.1 (0.7)	15.9 ± 10.5 11.1 (0.7)	0.896
P53%	Pre	8.7 ± 11.4 4.9 (1.3)	3.5 ± 4.8 1.9 (1.4)	0.580
	Post	3.9 ± 4.4 2.3 (1.1)	5.4 ± 7.3 3.2 (1.3)	0.886
NF-κB%	Pre	36.7 ± 21.3 29.6 (0.6)	52.7 ± 20.9 49.3 (0.4)	0.080
	Post	42.3 ± 31.7 37.6 (0.7)	50.7 ± 26.7 43.5 (0.5)	0.445
β-catenin%	Pre	100.0 ± 0.0 100.0 (0.0)	100.0 ± 0.0 100.0 (0.0)	1.000
	Post	100.0 ± 0.0 100.0 (0.0)	100.0 ± 0.0 100.0 (0.0)	1.000
Dysplastic Tissue				
Ki-67%	Pre	44.9 ± 26.4 37.6 (0.6)	42.9 ± 19.5 39.1 (0.5)	0.913
	Post	58.0 ± 26.0 50.1 (0.4)	58.2 ± 23.6 53.1 (0.4)	0.931
P53%	Pre	50.0 ± 24.2 44.8 (0.5)	37.5 ± 20.5 32.4 (0.5)	0.419
	Post	60.3 ± 27.8 52.4 (0.5)	51.8 ± 24.1 46.2 (0.5)	0.413
NF-κB%	Pre	69.7 ± 17.2 67.1 (0.2)	74.3 ± 15.0 72.6 (0.2)	0.445
	Post	74.3 ± 21.5 67.8 (0.3)	81.4 ± 13.5 80.3 (0.2)	0.383
β-catenin%	Pre	100.0 ± 0.0 100.0 (0.0)	98.6 ± 5.3 98.4 (0.1)	0.743
	Post	100.0 ± 0.0 100.0 (0.0)	100.0 ± 0.0 100.0 (0.0)	1.000

Data are shown as mean ± SD (first line) and geometric mean (coefficient of variation (CV; second line). Pre indicates the analysis at baseline, before supplementation; Post indicates the analysis after the prolonged supplementation with active or placebo arms. The tissue biomarkers expression levels in normal and dysplastic tissues were estimated semi-quantitatively as percentage of positive cells. Abbreviations: Ki-67, protein encoded by the MKI67 gene; P53, protein encoded by the TP53 gene; NF-κB, nuclear factor kappa-light-chain-enhancer of activated B cells; β-catenin, protein encoded by the CTNNB1 gene.

## Data Availability

Data may be made available for collaborative studies upon reasonable request to the corresponding author.

## Supplementary Materials

The following are available online at https://www.mdpi.com/article/10.3390/ijms222011024/s1.

## Abbreviations

MDPI	Multidisciplinary Digital Publishing Institute
DOAJ	Directory of Open Access Journals
TLA	three letter acronym
LD	linear dichroism

## References

[1] Ferlay J., Colombet M., Soerjomataram I., Dyba T., Randi G., Bettio M., Gavin A., Visser O., Bray F. (2018). Cancer incidence and mortality patterns in Europe: Estimates for 40 countries and 25 major cancers in 2018. Eur. J. Cancer.

[2] Keum N., Giovannucci E. (2019). Global burden of colorectal cancer: Emerging trends, risk factors and prevention strategies. Nat. Rev. Gastroenterol. Hepatol..

[3] O’Shaughnessy J.A., Kelloff G.J., Gordon G.B., Dannenberg A.J., Hong W.K., Fabian C.J., Sigman C.C., Bertagnolli M.M., Stratton S.P., Lam S. (2002). Treatment and prevention of intraepithelial neoplasia: An important target for accelerated new agent development. Clin. Cancer Res..

[4] Terzić J., Grivennikov S., Karin E., Karin M. (2010). Inflammation and colon cancer. Gastroenterology.

[5] Rothwell P.M., Wilson M., Elwin C.E., Norrving B., Algra A., Warlow C.P., Meade T.W. (2010). Long-term effect of aspirin on colorectal cancer incidence and mortality: 20-year follow-up of five randomised trials. Lancet..

[6] Jakszyn P., Cayssials V., Buckland G., Perez-Cornago A., Weiderpass E., Boeing H., Bergmann M.M., Vulcan A., Ohlsson B., Masala G. (2020). Inflammatory potential of the diet and risk of colorectal cancer in the European Prospective Investigation into Cancer and Nutrition study. Int. J. Cancer..

[7] Continuous Update Project Report Food, Nutrition, Physical Activity, and the Prevention of Colorectal Cancer. http://www.aicr.org/assets/docs/pdf/reports/Second_Expert_Report.pdf.

[8] Pricci M., Girardi B., Giorgio F., Losurdo G., Ierardi E., Di Leo A. (2020). Curcumin and Colorectal Cancer: From Basic to Clinical Evidences. Int. J. Mol. Sci..

[9] Howells L., Malhotra Mukhtyar R., Theofanous D., Pepper C., Thomas A., Brown K., Khan S. (2021). A Systematic Review Assessing Clinical Utility of Curcumin with a Focus on Cancer Prevention. Mol. Nutr. Food Res..

[10] Cuomo J., Appendino G., Dern A.S., Schneider E., McKinnon T.P., Brown M.J., Togni S., Dixon B.M. (2011). Comparative absorption of a standardized curcuminoid mixture and its lecithin formulation. J. Nat. Prod..

[11] Bresciani L., Favari C., Calani L., Francinelli V., Riva A., Petrangolini G., Allegrini P., Mena P., Del Rio D. (2020). The Effect of Formulation of Curcuminoids on Their Metabolism by Human Colonic Microbiota. Molecules.

[12] Teng C.F., Yu C.H., Chang H.Y., Hsieh W.C., Wu T.H., Lin J.H., Wu H.C., Jeng L.B., Su I.J. (2019). Chemopreventive Effect of Phytosomal Curcumin on Hepatitis B Virus-Related Hepatocellular Carcinoma in A Transgenic Mouse Model. Sci. Rep..

[13] Panahi Y., Kianpour P., Mohtashami R., Jafari R., Simental-Mendía L.E., Sahebkar A. (2017). Efficacy and Safety of Phytosomal Curcumin in Non-Alcoholic Fatty Liver Disease: A Randomized Controlled Trial. Drug Res..

[14] Szymanski M.C., Gillum T.L., Gould L.M., Morin D.S., Kuennen M.R. (2018). Short-term dietary curcumin supplementation reduces gastrointestinal barrier damage and physiological strain responses during exertional heat stress. J. Appl. Physiol..

[15] Hu S., Belcaro G., Dugall M., Peterzan P., Hosoi M., Ledda A., Riva A., Giacomelli L., Togni S., Eggenhoffner R. (2018). Interaction study between antiplatelet agents, anticoagulants, thyroid replacement therapy and a bioavailable formulation of curcumin (Meriva®). Eur. Rev. Med. Pharmacol. Sci..

[16] Ali H.M., Almagribi W., Al-Rashidi M.N. (2016). Antiradical and reductant activities of anthocyanidins and anthocyanins, structure-activity relationship and synthesis. Food Chem..

[17] Wang L.S., Stoner G.D. (2008). Anthocyanins and their role in cancer prevention. Cancer Lett..

[18] Dharmawansa K.V.S., Hoskin D.W., Rupasinghe H.P.V. (2020). Chemopreventive Effect of Dietary Anthocyanins against Gastrointestinal Cancers: A Review of Recent Advances and Perspectives. Int. J. Mol. Sci..

[19] Wang X., Yang D.Y., Yang L.Q., Zhao W.Z., Cai L.Y., Shi H.P. (2019). Anthocyanin Consumption and Risk of Colorectal Cancer: A Meta-Analysis of Observational Studies. J. Am. Coll Nutr..

[20] Riva A., Togni S., Franceschi F., Kawada S., Inaba Y., Eggenhoffner R., Giacomelli L. (2017). The effect of a natural, standardized bilberry extract (Mirtoselect®) in dry eye: A randomized, double blinded, placebo-controlled trial. Eur. Rev. Med. Pharmacol. Sci..

[21] Hoggard N., Cruickshank M., Moar K.M., Bestwick C., Holst J.J., Russell W., Horgan G. (2013). A single supplement of a standardised bilberry (*Vaccinium myrtillus* L.) extract (36 % wet weight anthocyanins) modifies glycaemic response in individuals with type 2 diabetes controlled by diet and lifestyle. J. Nutr. Sci..

[22] Mazzolani F., Togni S., Franceschi F., Eggenhoffner R., Giacomelli L. (2017). The effect of oral supplementation with standardized bilberry extract (Mirtoselect®) on retino-cortical bioelectrical activity in severe diabetic retinopathy. Minerva Oftalmol..

[23] Sharma R.A., Steward W.P., Gescher A.J., Aggarwal B.B., Surh Y.J., Shishodia S. (2007). Pharmacokinetics and Pharmacodynamics of Curcumin. The Molecular Targets and Therapeutic Uses of Curcumin in Health and Disease.

[24] Vareed S.K., Kakarala M., Ruffin M.T., Crowell J.A., Normolle D.P., Djuric Z., Brenner D.E. (2008). Pharmacokinetics of curcumin conjugate metabolites in healthy human subjects. Cancer Epidemiol. Biomark. Prev..

[25] Sarkar F.H., Li Y. (2008). NF-kappaB: A potential target for cancer chemoprevention and therapy. Front. Biosci..

[26] Rajitha B., Belalcazar A., Nagaraju G.P., Shaib W.L., Snyder J.P., Shoji M., Pattnaik S., Alam A., El-Rayes B.F. (2016). Inhibition of NF-κB translocation by curcumin analogs induces G0/G1 arrest and downregulates thymidylate synthase in colorectal cancer. Cancer Lett..

[27] Jobin C., Bradham C.A., Russo M.P., Juma B., Narula A.S., Brenner D.A., Sartor R.B. (1999). Curcumin blocks cytokine-mediated NF-kappa B activation and proinflammatory gene expression by inhibiting inhibitory factor I-kappa B kinase activity. J. Immunol..

[28] Thomasset S., Berry D.P., Cai H., West K., Marczylo T.H., Marsden D., Brown K., Dennison A., Garcea G., Miller A. (2009). Pilot study of oral anthocyanins for colorectal cancer chemoprevention. Cancer Prev. Res..

[29] Perez-Vizcaino F., Duarte J., Santos-Buelga C. (2012). The flavonoid paradox: Conjugation and deconjugation as key steps for the biological activity of flavonoids. J. Sci. Food Agric..

[30] Shimoi K., Saka N., Nozawa R., Sato M., Amano I., Nakayama T., Kinae N. (2001). Deglucuronidation of a flavonoid, luteolin monoglucuronide, during inflammation. Drug Metab. Dispos..

[31] Anand P., Kunnumakkara A.B., Newman R.A., Aggarwal B.B. (2007). Bioavailability of curcumin: Problems and promises. Mol. Pharm..

[32] Marczylo T.H., Verschoyle R.D., Cooke D.N., Morazzoni P., Steward W.P., Gescher A.J. (2007). Comparison of systemic availability of curcumin with that of curcumin formulated with phosphatidylcholine. Cancer Chemother. Pharmacol..

[33] Allegri P., Mastromarino A., Neri P. (2010). Management of chronic anterior uveitis relapses: Efficacy of oral phospholipidic curcumin treatment. Long-term follow-up. Clin. Ophthalmol..

[34] Franceschi F., Feregalli B., Togni S., Cornelli U., Giacomelli L., Eggenhoffner R., Belcaro G. (2016). A novel phospholipid delivery system of curcumin (Meriva®) preserves muscular mass in healthy aging subjects. Eur. Rev. Med. Pharmacol. Sci..

[35] Cai H., Thomasset S.C., P-Berry D., Garcea G., Brown K., Steward W.P., Gescher A.J. (2011). Determination of anthocyanins in the urine of patients with colorectal liver metastases after administration of bilberry extract. Biomed. Chromatogr..

[36] Carroll R.E., Benya R.V., Turgeon D.K., Vareed S., Neuman M., Rodriguez L., Kakarala M., Carpenter P.M., McLaren C., Meyskens F.L. (2011). Phase IIa Clinical Trial of Curcumin for the Prevention of Colorectal Neoplasia. Cancer Prev. Res..

[37] Mahale J., Singh R., Howells L.M., Britton R.G., Khan S.M., Brown K. (2018). Detection of Plasma Curcuminoids from Dietary Intake of Turmeric-Containing Food in Human Volunteers. Mol. Nutr. Food Res..

[38] Irving G.R., Howells L.M., Sale S., Kralj-Hans I., Atkin W.S., Clark S.K., Britton R.G., Jones D.J., Scott E.N., Berry D.P. (2013). Prolonged biologically active colonic tissue levels of curcumin achieved after oral administration—A clinical pilot study including assessment of patient acceptability. Cancer Prev. Res..

[39] Pal A., Sung B., Prasad B.A.B., Schuber P.T., Prasad S., Aggarwal B.B., Bornmann W.G. (2014). Curcumin glucuronides: Assessing the proliferative activity against human cell lines. Bioorg. Med. Chem..

[40] Hsu S.M., Raine L., Fanger H. (1981). A comparative study of the peroxidase–antiperoxidase method and an avidin–biotin complex method for studying polypeptide hormones with radioimmunoassay antibodies. Am. J. Clin. Pathol..

