# Supplementary material for: A Presurgical Study of Curcumin Combined with Anthocyanin Supplements in Patients with Colorectal Adenomatous Polyps
Source: Int J Mol Sci. 2021 Oct 13;22(20):11024. doi: 10.3390/ijms222011024 (PMC8539981; doi:10.3390/ijms222011024)
Supplement: Supplementary file 1 [file ijms-22-11024-s001.zip › ijms-1393016-supplementary.pdf]

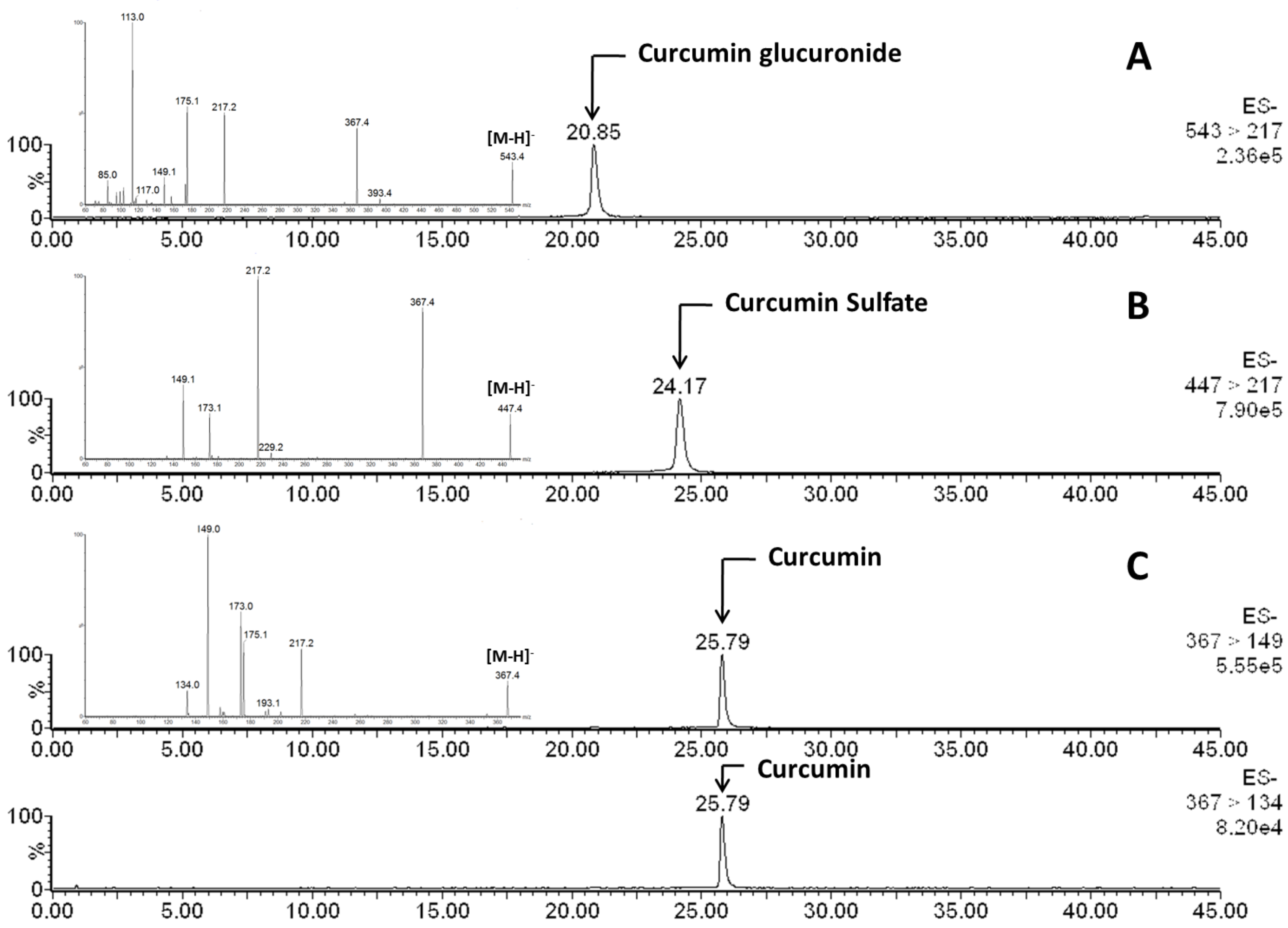

Supplementary Figure S1. A representative set of LC-ESI-MS/MS selected reaction monitoring (SRM) chromatograms obtained for the analysis of synthetic standards of (**A**) curcumin glucuronide, (**B**) curcumin sulfate, (**C**) curcumin. The amount injected was 1000 fmol on column for each analyte. For parent curcumin, the SRM channel 367→149 m/z was used as the quantifier channel and the 367→134 m/z transition, shown in the bottom chromatogram, was used as the qualifier channel to confirm the identity. Also shown, as inserts on each chromatogram (A-C) are the negative ESI-MS/MS collision induced

dissociation product ion spectra to verify the identity. Samples were analysed using the system and conditions described in Section 4.4 of the Materials and Methods.

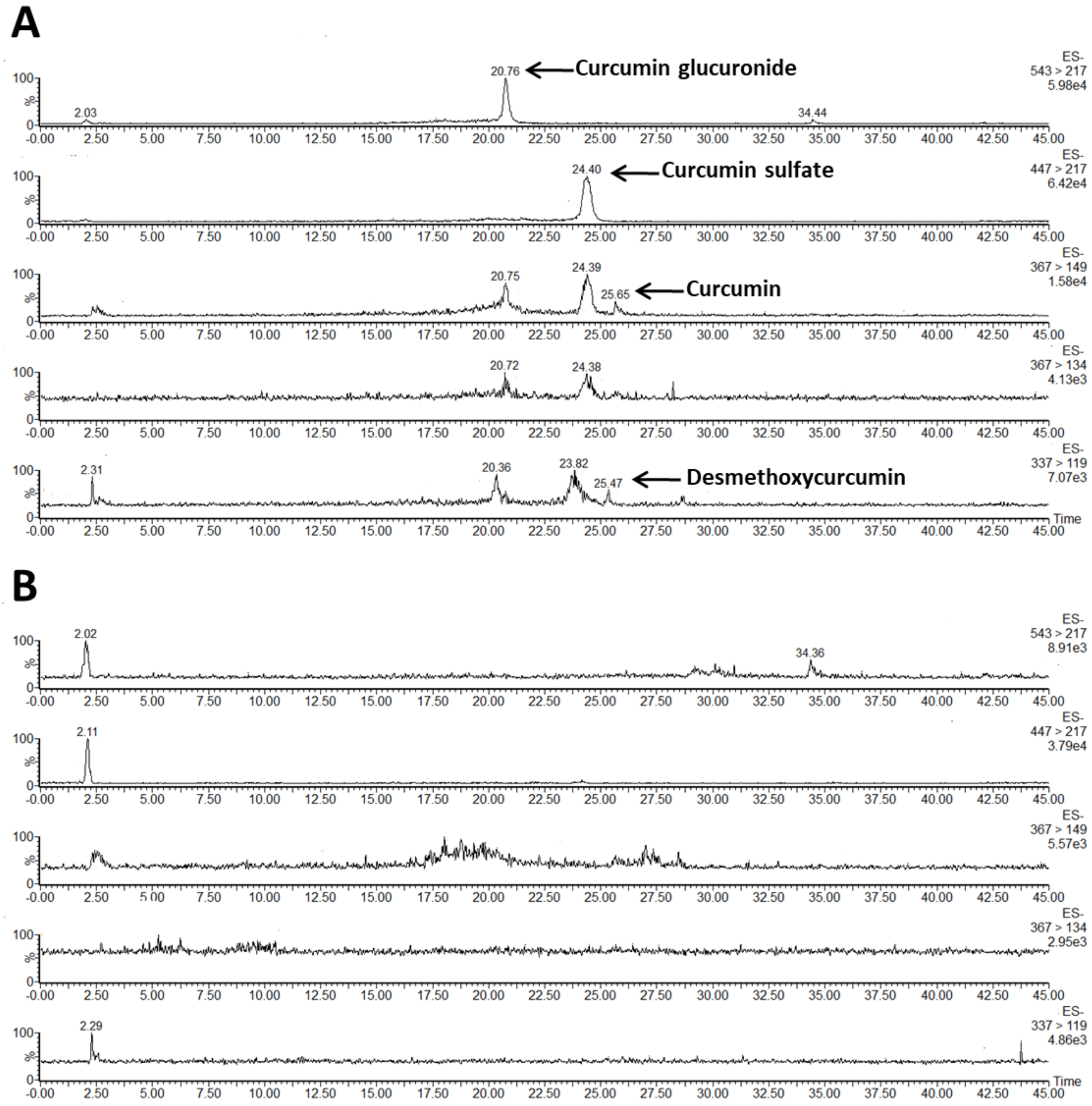

Supplementary Figure S2. A representative set of LC-ESI-MS/MS SRM chromatograms obtained for the analysis of (**A**) a plasma sample from a patient randomised to the active arm of the trial that was taken 1 h after the last dose of curcumin and (**B**) a control blank plasma sample where no curcumin or metabolite peaks were detected. The samples were analysed in negative ESI mode with SRM for the $[M\text{-}H]^-$ ion transitions of curcumin and metabolites: curcumin glucuronide 543 to 217 *m/z*, curcumin sulphate 447 to 217*m/z*, curcumin 367 to 149 *m/z*, 367 to 134 *m/z* and desmethoxycurcumin 337 to 119 *m/z*. The additional transition 367 to 134 m/z was used as the qualifier channel to confirm the identity of the curcumin peak. Samples were processed and analysed using the system and conditions described in Section 4.4 of the Materials and Methods.
